# Heat Shock Causes Lower *Plasmodium* Infection Rates in *Anopheles albimanus*

**DOI:** 10.3389/fimmu.2021.584660

**Published:** 2021-06-24

**Authors:** Renaud Condé, Erika Hernandez-Torres, Fabiola Claudio-Piedras, Benito Recio-Tótoro, Krystal Maya-Maldonado, Victor Cardoso-Jaime, Humberto Lanz-Mendoza

**Affiliations:** ^1^ Centro de Investigaciones Sobre Enfermedades Infecciosas, Instituto Nacional de Salud Pública, Cuernavaca, Mexico; ^2^ Instituto de Biotecnología, Universidad Nacional Autónoma de México, Cuernavaca, Mexico

**Keywords:** *Anopheles albimanus*, *Plasmodium berghei*, heat shock, immunity, infection resistance

## Abstract

The immune response of *Anopheles* mosquitoes to *Plasmodium* invasion has been extensively studied and shown to be mediated mainly by the nitric oxide synthase (NOS), dual oxidase (DUOX), phenoloxidase (PO), and antimicrobial peptides activity. Here, we studied the correlation between a heat shock insult, transcription of immune response genes, and subsequent susceptibility to *Plasmodium berghei* infection in *Anopheles albimanus*. We found that transcript levels of many immune genes were drastically affected by the thermal stress, either positively or negatively. Furthermore, the transcription of genes associated with modifications of nucleic acid methylation was affected, suggesting an increment in both DNA and RNA methylation. The heat shock increased PO and NOS activity in the hemolymph, as well as the transcription of several immune genes. As consequence, we observed that heat shock increased the resistance of mosquitoes to *Plasmodium* invasion. The data provided here could help the understanding of infection transmission under the ever more common heat waves.

## Introduction

Insects are commonly stressed by pathogens (1), toxic compounds, dietary factors, temperature (2), and hypoxia (3). These stressors share commonalities in their effects on the molecular components of cells, and therefore much of the transcriptional response they elicit is conserved (4). There is significant crosstalk in the signalling cascades that regulate each of the various stress-specific transcription factors (5, 6) and so any stressor has the potential to, and generally does, affect the transcription of genes that are not directly related to the specific stress applied (7). When microorganisms challenge insects, molecular patterns on the pathogen trigger alterations in transcription *via* Toll, IMD, and the JAK-STAT pathway, ultimately activating the Rel1, Rel2, and STAT transcription factors. These transcriptional factors control the expression of hundreds of genes, including many immune response genes. The immune response has been shown to be intertwined with the general stress response in several insect models, and a number of genes activated by pathogen invasion are not directly linked to anti-pathogenic functions. For instance, LPS injection of the *Tribolium castaneum* beetle also induces heat shock stress response genes (HSP 68 and HSP 27) and hypoxia-inducible gene transcription (7). Conversely, in the same insect, heat shock triggers the transcription of the immune genes *tlr6*, *pgrp2*, *defensin1*, and *defensin2* (8). In *Galleria mellonella* larvae, heat shock alters the expression of antimicrobial peptide genes. For example, apoLp-III gene expression is transiently inhibited after heat shock but, when an infection occurs 96 h after the heat shock, it is induced at higher levels than in the absence of prior heat shock (9). Some *Drosophila melanogaster* immune genes are regulated by the heat shock factor, and in fact, most Heat shock Factor Binding Sites in the genome are found in Non-Heat shock Genes (10). The regulatory cascades of Heat shock Factor 1 and NF-kB share some components, such as Droj2 (HSP40) that functions downstream of or directly regulates Relish and DNAJA3 which are required for IκB phosphorylation (11).

Interestingly, heat shock-mediated immune activation can be transgenerational, hence affecting immune homeostasis over a long period of time (12). Upon stress, the beetle *Nicrophorus vespilloides* initiates a transposon-mediated genomic reorganization of its immune genes, suggesting that this reorganization might constitute a common pathway to enhance survival during protein damage (13). This reorganization could occur because stress or infection mobilizes HSP 90 protein, impairing its mutation-dampening function (14). Aside from heat shock direct transcriptional effects, or genomic reorganization, the temperature also affects the function of enzymes that modify genomic DNA such as the Ten eleven translocation (TET) dioxygenase, providing another level of transcriptional regulation (15).

HSP70 also protects the midgut of *Aedes aegypti* mosquitoes against the dramatic increase in body temperature after ingesting a hot blood meal (16), considering that the mosquito ingests its own weight of blood at 37°C (17). Though immune signaling pathways of anopheline mosquitoes have been scrutinized in great detail, little is known about their interactions with hypoxia (HIF) and heat shock factor (HSF) mediated transcriptional regulation. The blood-feeding of female mosquitoes implies that a heat shock is produced in the midgut and that HSP70 proteins are transiently expressed (16). In anopheline mosquitoes, Toll and Imd pathways show a certain amount of cross signaling (18); opening the possibility of a cooperative effect of bacterial infection response and *Plasmodium* mediated response, as shown in *Anopheles gambiae* by Ramirez et al. (19). The gut tissues of hematophagous insects are subjected to large temperature changes when feeding. As a consequence, HSP 82, HSP 90, and HSP 105 of *A. gambiae* are up-regulated during blood-feeding (20, 21). Later, *ex vivo* analysis of the *A. gambiae* HSC70B promoter revealed that the transcription was influenced by immune activation (5), proving the existence of a cross-talk between immune and heat shock activation cascades. In *Anopheles stephensi*, the rearing temperature alters the transcription levels of immune effectors differentially. While the nitric oxide synthase (NOS) expression peaked at 30°C, the humoral melanization, phagocytosis, and defensin expression were maximum at 18°C (22), though the melanization reaction has been described as a minor factor in resistance to *P. falciparum* (23). In general, phenoloxidase (PO) and NO have been implicated in the immune response to *Plasmodium* infection in *Anopheles.* In *A. stephensi*, the metabolites of NOS activity increase nitric oxide concentrations and subsequent ookinete death (24). The enzymatic cleavage regulating NOS activity is temperature-dependent (25). Some heat shock proteins (HSPs), such as *A. gambiae* HSC70B have shown antiviral activities capable of suppressing *O’nyong’nyong* viral infection (26). In turn, this observation opens the possibility that the HSPs could affect the microorganisms directly, once released in the hemolymph. In particular, heat shock triggers a general damage response in mosquitoes, including immune gene transcription. Though *P. berghei* does not constitute a natural *A. albimanus* parasite, this model has proven an invaluable tool for the study of the immune response of this insect. Here, we use this model to investigate the effects of heat shock on the expression of immune-related genes and the subsequent effect on susceptibility to infection.

## Materials and Methods

### Mosquito Rearing and Infection With *P. berghei*

A *Plasmodium-*susceptible strain of *A. albimanus* females (27) were obtained from the insectary of the National Institute of Public Health (INSP) in Cuernavaca, Mexico. Mosquitoes were bred under a 12:12 photoperiod at 28°C and 70–80% relative humidity. At four-days post-emergence, mosquitoes were infected with *P. berghei* ANKA strain expressing the green fluorescent protein (GFP) (28) (kindly donated by Robert E. Sinden, Imperial College, U.K.). Ookinetes were produced by culturing gametocyte-infected mouse blood, as described previously (29). Groups of 300 female mosquitoes were fed for 1 h using artificial membrane feeders with: (i) mouse blood + GFP ookinetes (infected group, with approximately 900 ookinetes per μl), or (ii) mouse blood only (control group). Unfed mosquitoes were removed, and the engorged ones maintained at 21°C to allow for parasite invasion and interaction with the mosquito midgut. Three experimental repetitions were performed.

### Mosquito Heat Shock

Since in *Drosophila* HSF binding reaches a maximum level following a 30-minute heat shock at 36.5°C (30), and mosquitoes endure a thermic shock while feeding on mammals blood (16), we exposed the mosquitoes to 30 min of 37°C heat shock, with 80% humidity and availability of 10% sugared water. In nature, the blood feeding is concomitant to the heat shock in naturally infected mosquitoes, and lead to the infection of the mosquito (16). Since the objective of our experiments is to test the effect of abiotic stress on the susceptibility to *P. berghei* infection of *A. albimanus*, we challenged them thermally 6 h before the infective blood meal.

### Mosquito Protein Extract Preparation for Acrylamide Electrophoresis

About 10 mosquitoes per sample were homogenized in 100 µl of lysis solution (8 M urea, 2 M thiourea, 1% Chaps, 13 mM DTT, and 4 µl of protease inhibitor cocktail (Sigma, P2714)). The resulting solution was cleared by 15 min centrifugation at 14,000*g* and 4°C. The total protein content of the supernatant was determined according to Lowry et al. (31). Samples were obtained before the heat shock, immediately after, and at 2 and 6 h after 30 min of exposure at 37°C.

### Acrylamide Electrophoresis and Western Blot Assay of the Mosquito Protein Extracts

Some 25 μg of mosquito protein extract were separated in SDS-PAGE (10% acrylamide) and transferred to Immobilon-P membrane. The protein transfer on the membrane was assessed by Ponceau S (P7170 Sigma-Aldrich) staining. For the Hsp-70 immunodetection, anti-Hsp70 monoclonal antibody [3A3] Thermo Scientific MA3-006 was used as primary antibody at a final dilution of 1:1,000, and goat anti-mouse-IgG-horseradish peroxidase (Abcam) diluted 1:1,000 as secondary antibody. Development of immunoblots was performed with an ECL kit from Amersham. Fluorescence was developed on a Kodak BioMax ML-2 film for capturing chemiluminescent data (Catalog Number Z370428) using Kodak Developer (Catalog Number P7042) and Kodak Fixer (Catalog Number P7167).

### Mosquito Hemolymph Collection

Hemolymph was obtained by perfusion from 30 control and heat-shocked mosquitoes (at six hours post-heat shock), and 24-hours post-*P. berghei* infection (30-hours post-heat shock) as described elsewhere (32). Three experimental repetitions were performed.

### Mosquito RNA Extraction

Total RNA from 10 whole female mosquitoes (without head) and midguts only, were obtained by Trizol method (Invitrogen) and then re-purified using RNA Clean-Up Kit (Zimo Research). cDNA was synthesized by reverse transcription using 1 µg of RNA, 100 ng of oligonucleotide dT, and 200 U of the enzyme reverse transcriptase RNase H-SuperScript II (Gibco BRL). Three experimental repetitions were performed, each counting three samples per conditions.

### RT-qPCR Amplification

The amplification of genes of the *An. albimanus* immune response was carried out with previously recovered genetic material. Specific primers were used for each gene (**Table 1**).

**Table 1:** Oligonucleotides used to amplify the mRNA transcripts of *A. albimanus* genes.

*ppo1* F	5’-GGCGGACCAAATCAAGCAG-3’
*ppo1* R	5’-CGATTGCCCGATTCGTCAAC-3’
*tet 2 F*	5’-TCCTCCGATCCGAGGATCAGGT-3’
*tet 2* R	5’-GTACCTTGCTGTTGCTGGGCA-3’
*dnmt2* F	5’-GAGCCATCTTTTCCGATTCGTC-3’
*dnmt2* R	5’-GAGCCATCTTTTCCGATTCGTC-3’
CECA-R	5’-ATTTGCCAAGTGCCTTCAC-3’
CECA-F	5’-AGTGGACGCTGGTTTTCTCAAG-3’
Gam F	5’-CGCTTATGCTTCGACTTGC-3’
Gam R	5’-AATCATCGTCTGACCATCGC-3’
GNBPA F	5´-CACTCGATACGGAGTCGGC-3´
GNBPA R	5’-AACTAATCTGGGCTCATCGTG-3’
*duox* F	5’-CTCTCTCTGTTGCAGAATCCAG-3’
*duox* R	5’-TGGTGTGAGATGGTTATCGACT-3’
*hsp70* F	5’-CCAGCATGGAAAGGTGGAGA-3’
*hsp70* R	5’-CCATCCATCAGGGCGTCAAT-3’
*frep3* F	5’-CAGTGCGTGTCGTGCAAT-3’
*frep3* R	5’-AACCGTTTGAGAATCTGTAGCA-3’
S7 F	5’-AACAACAAGAAGGCCATCGTC-3’
S7 R	5’-GGCTTGGGCAGAATACGA-3’

ppo1, Prophenol oxidase 1; tet2, ten-eleven translocation methylcytosine dioxygenases; dnmt2, DNA methyltransferase 2; duox, Dual oxidase; hsp70, heat shock protein 70; frep3, Fibrinogen related protein 3, cecropin, gambicin and GNBP4.

The samples were run in a real-time thermal cycler (viiA7; Applied Biosystems) under optimal running conditions, according to the manufacturer’s recommendations. Samples were incubated at 60°C in a master mix containing SYBR Green (Maximum SYBR Green/Rox qPCR Master Mix; Thermo Scientific), primers, and cDNA of each of the samples, set to a volume of 20 μl with water free of nucleases (Thermo Scientific). The relative expression was quantified by normalizing the expression of immune response genes with the S7 ribosomal gene.

Assays were performed three times in different batches of 10 mosquitoes and three times in different batches of five mosquitoes’ midguts. The control and experimental tests were made at the same time. For real-time PCR, 2.5 µl of cDNA was used in SYBR Green I Kit (Applied Biosystems) following the kit instructions. The primers used are described in **Table 1**. The fold changes in expression were calculated using the comparative “delta delta Ct” (Ct) method against the blood-fed control (33) using three replicates per sample. Three independent experiments were done. The data represents the average fold-change relative to the control group. The amplification efficiency was similar between the test and control genes.

### Phenoloxidase (PO) Activity

PO activity was measured as described (34). Three pools of 30 female mosquitoes were macerated and centrifuged at 10,000*g* for 10 min at 4°C. L-DOPA was used as the substrate for PO, which is transformed into the dye dopachrome. Auto-oxidation controls (L-DOPA only) and blanks (macerated mosquitoes) were included. PO activity was measured every minute for 30 min at 490 nm in a microplate reader (ELISA iMark, BIO-RAD).

### NO Quantification

Nitrites $\left({\text{NO}}_{2}^{-}\right)$ and nitrates $\left({\text{NO}}_{3}^{-}\right)$ were evaluated by the Griess assay (35). Pools of 30 female mosquitoes per treatment were macerated and centrifuged twice at 10,000*g* for 10 min at 4°C. Proteins were eliminated with ZnSO_4_. Nitrates were reduced into nitrites using VCl_3_ immediately followed by the addition of sulfanilamide and NED. The reaction was incubated for 15 min at R.T., and the absorbance was measured at 490 and 630 nm in a microplate reader.

### Statistical Analysis

Data were analyzed and graphed in Prism v6.01 statistical software. qPCR results were evaluated by one way ANOVA followed Tukey’s test (Whole body RT-qPCR), and unpaired t test with Welch’s correction (Midgut RT-qPCR). The infection parameters were analyzed through Mann–Whitney. Considering that three independent repetitions of the experiment were performed, we applied a log-like generalized lineal model with random effect to determine the difference in ookinete prevalence between control and heat shocked mosquitoes. We used the individual experimental repetitions as categorical variable, this with the objective of measuring the effect of the individual repetitions on the mean differences between the two conditions. PO and NO results were analysed by Student’s t-test comparing the heat shock and control groups for the non-infected and infected mosquitoes.

## Results

### Heat Shock Diminishes Infection of an *A. albimanus* Susceptible Strain

**Figure 1:**
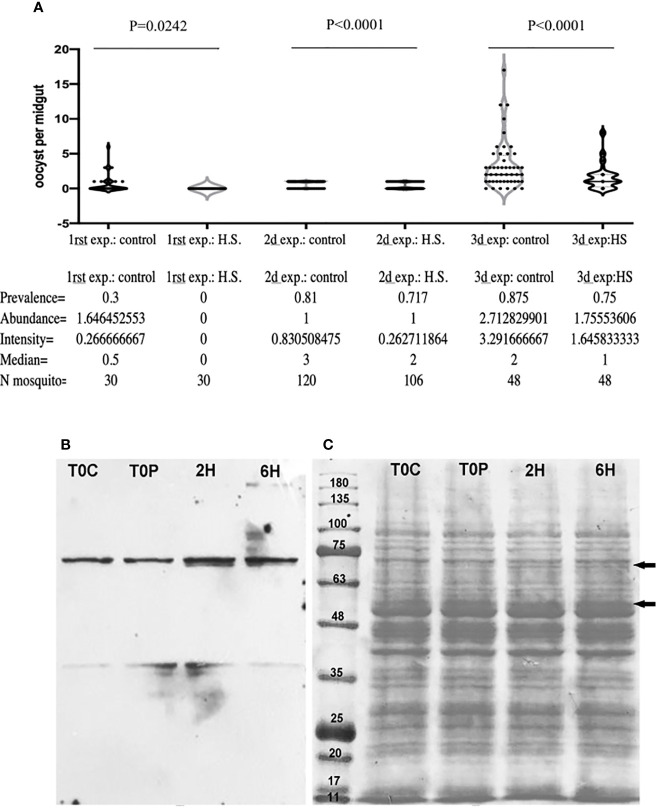
Effect of Heat shock on *P. berghei* infection in *A*. *albimanus.* **(A)** *A. albimanus* susceptible strain oocyst infection prevalence expressed as the ratio of infected mosquitoes over total mosquitoes sampled with (0.67 on average) and without previous heat shock (0.77 on average). **(B)** Western blot using the anti-HSP70 antibody of a 10% acrylamide gel of control 0, 2, and 6 h post-heat shock *A. albimanus* body protein extracts. **(C)** Protein profile of the *A. albimanus* body control T0C (control), T0P (Time zero post heat-shock), 2H (2 h post-heat shock), 6H (6 h post-heat shock). Black arrows indicate differential bands appearing in the ponceau red staining of protein profile of the 2 h post heat shock and 6 h post heat shock sample.

To assess the global effects of heat shock response on parasite development, female *A. albimanus* (susceptible strain) mosquitoes were heat-shocked at 37°C for half an hour, 6 h before infective blood-feeding (900 ookinetes/µl) in three separate experiments with three replicates. Oocyst numbers were assessed five days post blood meal. The prevalence and the intensity were significantly diminished in the heat-shocked mosquitoes (control 77.27%, n = 198 vs HS 67%, n = 184 with X^2^ = 4.7 and *p* = 0.0102). A median of three oocyst per mosquito midgut were found in control mosquitoes while a median of two oocysts per midgut were found in the heat shocked mosquitoes midguts (**Figure 1A**). A log-like generalized lineal model with random effect showed that oocyst prevalence is 83% smaller in the heat-shocked mosquitoes than in the control (95% IC 80–87%) with p = 0.00. The individual experiments performed (considering each experiment separately) do not affect the outcome of the analysis (P = 0.98).

Anti-HSP-70 western-blot analysis of the mosquito protein extracts shown in **Figure 1B** reveal an increase in expression of HSP 70 at both 2 and 6 h after heat shock. The protein profile of the mosquito’s midguts was altered by the treatment, demonstrating the impact of the heat stress on the mosquito cells 2 and 6 h post heat shock (**Figure 1C**, black arrows).

### Hemolymph and Body From Heat-Shocked *A. albimanus* Susceptible Strain Mosquitoes Present Higher Phenoloxidase Activity

Phenoloxidase activity has been previously described as an important factor limiting the *Anopheles* infection by *Plasmodium* (36). The effect of heat shock upon the enzyme activity could be key to understanding the reduction of the mosquito’s susceptibility to the parasite.

**Figure 2:**
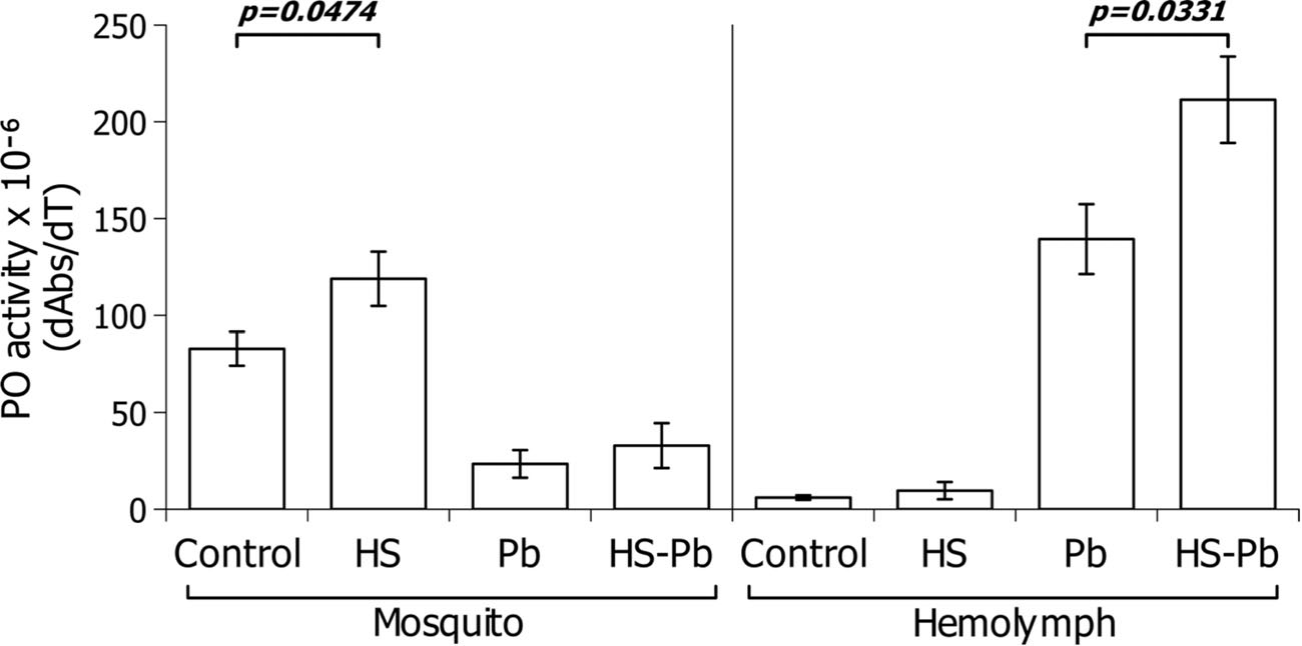
Effect of heat shock on the phenoloxidase response of female *A*. *albimanus* during a *P. berghei* infection. Phenoloxidase activity in macerated body extracts (right) and hemolymph (left). Control, Mosquitoes without heat shock; HS, heat shock mosquitoes; Pb, *Plasmodium berghei* fed mosquitoes. Data of three independent experiments were analyzed using Student’s t-test and are represented with their standard deviations.

Therefore, we measured the PO activity in the hemoplymph and full mosquito body during the heat shock. In three separate experiments, the hemolymph from 30 female *A. albimanus* susceptible strain that were heat-shocked at 37°C for 30 min was collected. The sampled hemolymph was obtained at 6 h post-heat shock, and 24 h post-*P. berghei* infection (30 h post-heat shock). Samples were tested for phenoloxidase activity using the colorimetric L-DOPA assay. As can be seen in **Figure 2**, phenoloxidase activity was altered by the heat shock regime. The heat-shock by itself did not increase the phenoloxidase activity significantly in the hemolymph. As shown previously (37, 38), *P. berghei* infection increased hemolymph phenoloxidase activity, mainly through enzymatic activation by proteolysis and secretion of prophenoloxidase. While heat shock alone did not increase hemolymph phenoloxidase activity, heat shock increased the hemolymph phenoloxidase activity (**Figure 2**) in the *P.berghei* infected mosquito hemolymph when compared to the heat-shocked non infected mosquitoes hemolymph and relative to non-heat shocked infected mosquitoes.

The phenoloxidase activity encountered in the whole mosquito body increased upon heat shock. However, when comparing both control/Pb and HS/HS Pb conditions, the activity diminishes upon infection. When considering the whole mosquito body mRNA, the heat shock did alter the PPO gene transcription at 6 h post-heat shock (**Figure 4**), and showed a tendency to diminish in the midgut (**Figure 5**). Considering that, in insects, the central organ of phenoloxidase production are the hemocytes (39) this result was expected.

### Hemolymph From Heat-Shocked *A. albimanus* susceptible Strain Mosquitoes Present Higher Nitric Oxide Concentration

**Figure 3:**
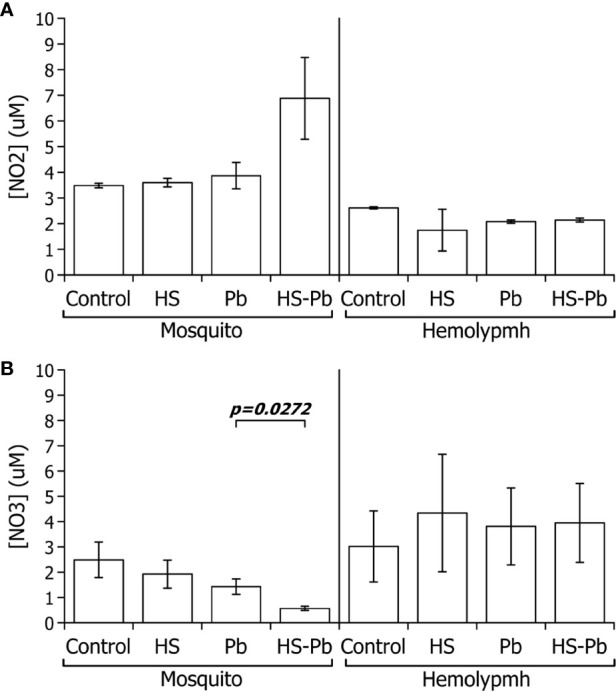
Effect of heat shock on the nitric oxide derivatives of *A. albimanus* during a *P. berghei* infection. **(A)** ${\text{NO}}_{2}^{-}$ production **(B)** ${\text{NO}}_{3}^{-}$ production. Nitric oxide derivatives present in the hemolymph and body of *A*. *albimanus* female mosquitoes upon *P. berghei* (Pb) infection and 6 h post 30 min at 37°C heat-shock (heat shock) and the respective control samples. Data of three independent experiments were analyzed using Student’s t-test.

Another key factor involved in the immune response of *A. albimanus* to *Plasmodium* infection occurs through NO synthesis (32). The only stable product of NO, which decays in seconds, is nitrite $\left({\text{NO}}_{2}^{-}\right)$, however, the more oxidized nitrate $\left({\text{NO}}_{3}^{-}\right)$ can also be produced. Therefore, to measure the total NO production, one must measure the total NOx derivatives. The${\text{NO}}_{2}^{-}$ found in the hemolymph of the heat-shocked mosquitoes diminished (**Figure 3A**) while ${\text{NO}}_{3}^{-}$ levels increased when compared with their control (**Figure 3B**), indicating that total NOx production was not changed. This may, however, indicate more oxidizing conditions in the hemolymph after heat shock. In whole body extracts, we observed that the combination of HS and infection increased${\text{NO}}_{2}^{-}$ production above either heat shock or infection alone (**Figure 3**).${\text{NO}}_{3}^{-}$ production in the body of mosquitoes was decreased by heat shock both in uninfected and those infected by *P. berghei*. From these results we suggest that *P. berghei* infection creates reducing conditions in the mosquito body that limits the full oxidation of nitrites into nitrates (**Figure 3B**).

### Heat Shock Response Affects Gene Transcription in the Whole Mosquito Body

**Figure 4:**
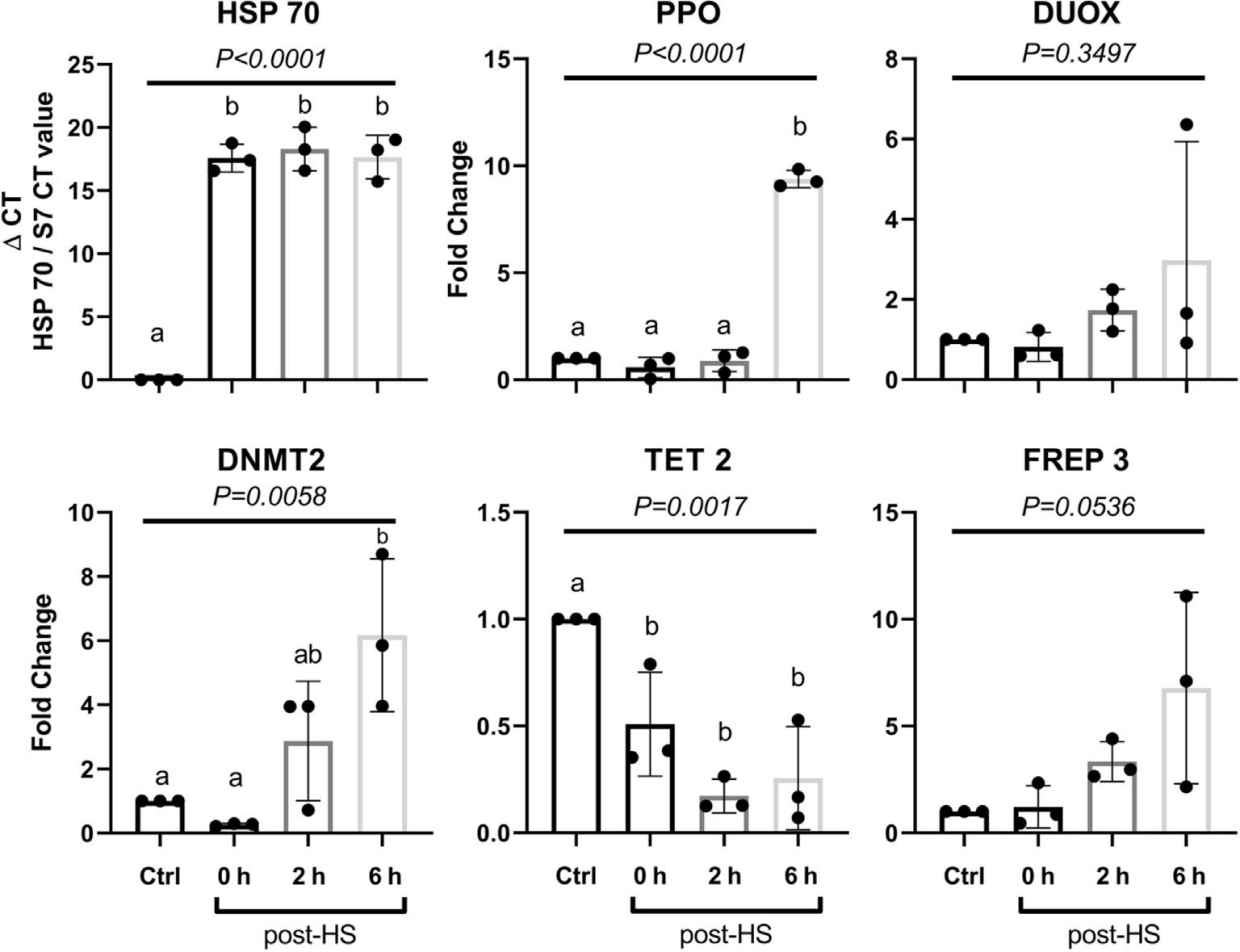
Gene expression at different times post-heat shock in whole body. Gene expression was evaluated immediately (0 h), at 2, and 6 h after 30 min of exposure at 37°C (heat shock) in whole mosquitoes body. Data are indicated as mean ± SD. Data of three independent experiments were analyzed using ANOVA-one way followed by Tukey’s test. Different letters indicate statistical significance.

To follow the transcription of inducible heat shock response, RT-qPCR was performed on female *An. albimanus* cDNA. Three independent experiments were performed with three groups of five mosquitoes per condition, per experiment, and the S7 gene cDNA was used to normalize the RT-qPCR. We observed an increase of *ppo* and *hsp70* transcripts upon heat shock. Decreases in *tet* gene transcription were observed immediately after heat shock while *dnmt2* increased two hours post-heat shock, and continued to increase thereafter. The *hsp70* gene showed a transcriptional upsurge after the heat shock, as expected. The transcription of effector molecules commonly considered central to the early response to *P. berghei* infection, such as *duox* and *ppo*, also increased following heat shock. *ppo* transcription increased significantly 6 h post-heat shock, with *duox* increasing slightly (**Figure 4**, **Supplementary Information**).

### Heat Shock Alters Antimicrobial Peptide Genes Transcription in the Mosquito Midgut

**Figure 5:**
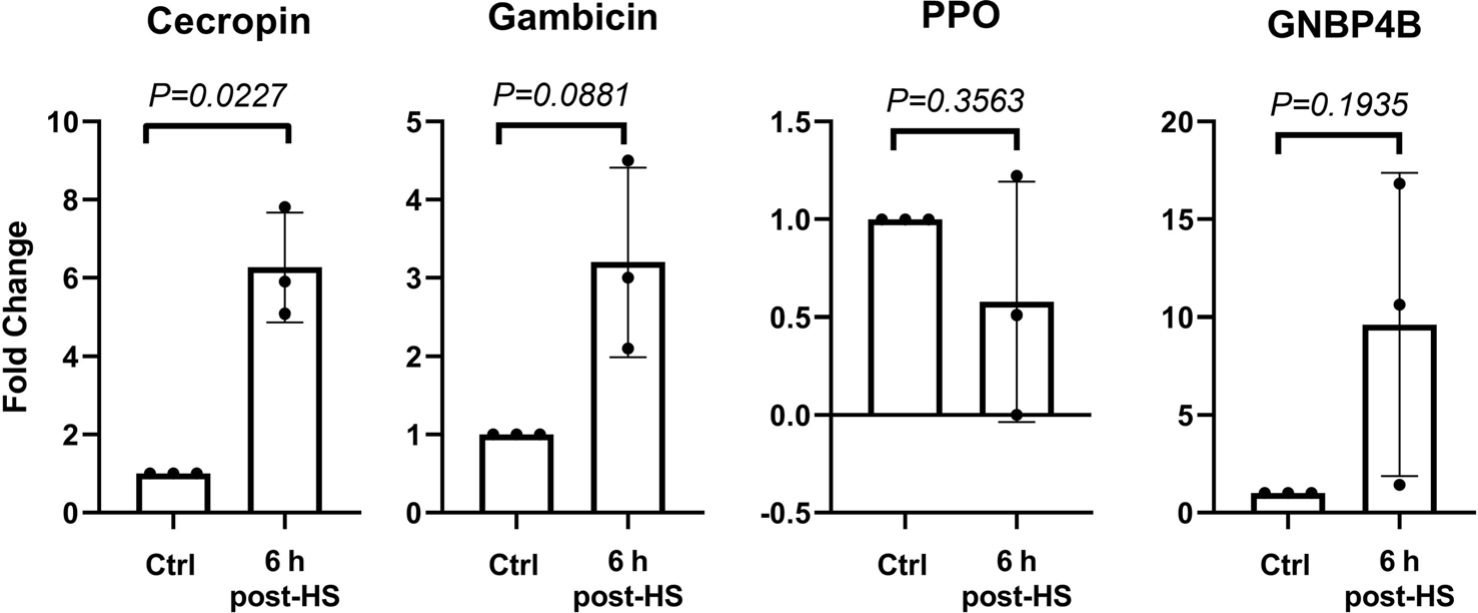
Gene expression at 6 h post heat shock in midguts. Gene expression was evaluated at 6 h after 30 min of exposure at 37°C (heat shock-HS) in midguts. Data of three independent experiments were analyzed using unpaired t test with Welch’s correction. Data are indicated as mean ± SD. Cecropin 6.2 ± 1.4, Gambicin 3.2 ± 1.2, PPO 0.5 ± 0.6, and GNBP4B 9.6 ± 7.7.

Control and heat-shocked mosquito midguts were extracted 6 h post-treatment, mRNA was extracted and cDNA synthetized. *Cecropin*, *gambicin*, *GNBP4B* and *ppo* genes were analyzed by RT-qPCR. Transcription of *ppo* was diminished at 6 h post-heat shock (**Figure 5**). This observation is in contradiction with whole body *ppo* transcription results. Inhibition of *ppo* transcription in the midgut could result from a specific sensitivity of this organ to heat shock or may involve a negative feedback loop resulting from proteolytic phenoloxidase activation. Transcription of the *cecropin*, *gambicin*, and *GNBP4B* genes increased upon heat shock (**Figure 5**, **Supplementary Information**) while *ppo* transcription slightly decreased, indicating again a crosstalk between heat shock and the immune response.

## Discussion

**Figure 6:**
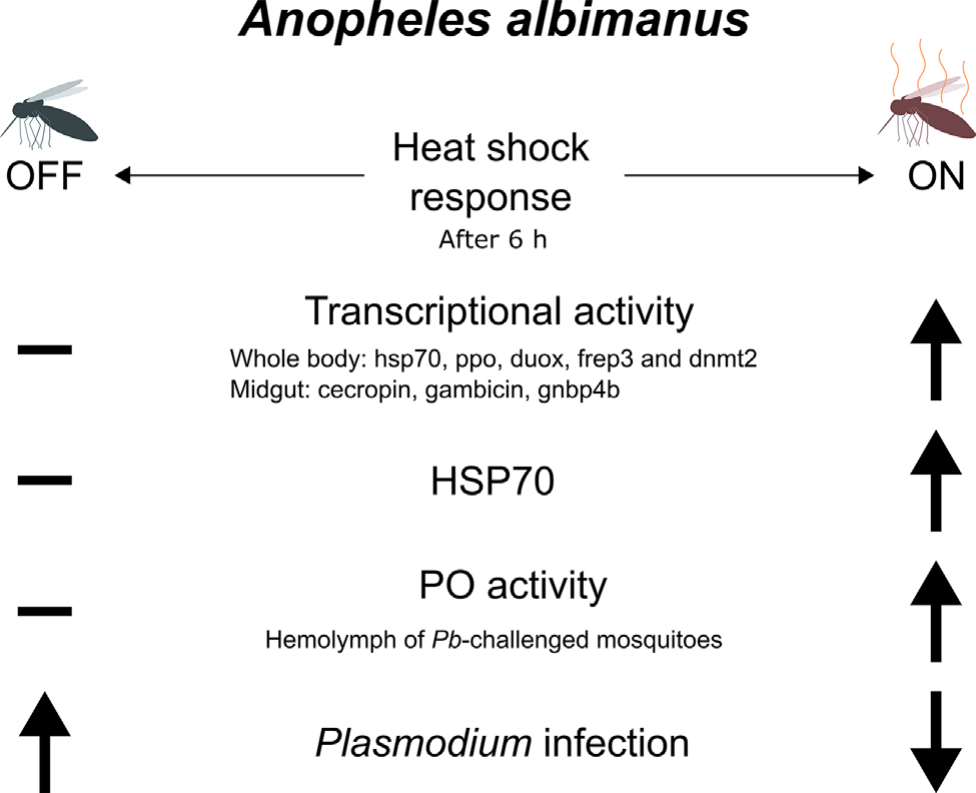
Schematic summary. After a 6 h of a 30 min heat shock at 37°C, several immune genes are upregulated in the mosquitoes. These include genes that have been shown responsible for limiting a *Plasmodium* infection such as *ppo*, *duox*, *frep3* in the whole mosquito, and antimicrobial peptides in the midgut. *hsp70* is also upregulated along with *dnmt2*, a gene responsible for the methylation of DNA and RNA in mosquitoes. This might indicate an increment in methylcytosines in the nucleic acids, which is also supported by the down regulation of the enzyme that removes these epigenetic marks, *tet2* (not shown). The PO activity also increases in the hemolymph of the *Plasmodium*-challenged and heat-shocked mosquitoes, although the total amount of NO produced did not changed (not shown). This overall response caused the mosquitoes to become less infected with the parasite. Only statistically significant results are portrayed.

Stress history drastically influences the mosquito immune system. The response to heat shock is generally characterized by heat shock protein expression. This response is, in the case of *A. gambiae*, protective from *O’nyong’nyong* virus infection (26). The experimental heat shock scheme chosen allowed us to track the effect of heat stress upon the mosquito immune system, although without a direct relation with the natural circumstances encountered by the mosquito in the wild, with the exception of the ever more common heat waves that are occurring worldwide due to climate change. Heat shock induces a state of increased resistance that lasts for at least 6 h. The heat shock possibly poises the immune system of the insect to respond faster and more intensively to the infection. For this to happen, the relevant changes must persist through the subsequent changes in the cellular environment. Methylation, demethylation, and other alterations to DNA and chromatin constitute a good theoretical mechanism for this ongoing physiological change. In mammals, the differentiation and specification of hematopoietic stem cells is mediated by *tet2* gene transcription. This gene codes for a methylcytosine dioxygenase that is required for activation of genes associated with differentiation (40, 41). In *A. albimanus*, the *tet2* gene could be involved in alteration of transcriptional activity upon *P. berghei* exposure (42). In this article, it was demonstrated that *P. berghei* infection of *A. albimanus* mosquitoes results in alterations in both DNA and mRNA methylation. The resulting methylation in turn alters the transcriptional pattern of the insect cells, suggesting longer term biological accommodation when challenged with *P. berghei* (42). In *Tribolium* beetles, general stress conditions do also lead to alterations in DNA methylation, allowing for wide spread transcriptional reprogramming (15). In insects, heat shock leads to substantial changes in the transcription, particularly in the expression of the Bt DNMT2 (DNA methyltransferase) gene, a part of the DNA methylation system. In the white fly *Bemisia tabaci* DNMT3 inhibition leads to an increase in heat susceptibility (43). Here, we observed that when aseptic stress in the form of heat shock is applied to *A. albimanus*, it enhances resistance to *P. berghei* infection. After heat shock, the activity of oxidative enzymes present in the mosquito hemolymph increased, potentially providing an explanation for the resistance observed (**Figure 6**). When analyzing the effect of heat shock on immunity gene transcription, we observed that genes related to DNA methylation modification (*tet*, *dnmt2*) showed the largest effect. In summary, heat shock alters the expression of many genes and induces the activation of phenoloxidase enzyme as well as increasing transcription of its gene. Infection also increased the activation of phenoloxidase.

The heat shock had little effect on ${\text{NO}}_{2}^{-}$ production, a species resulting from oxidation of the highly reactive NO. A slight increase in${\text{NO}}_{3}^{-}$ production was detected. Altogether, the results obtained demonstrate that immune alteration induced by heat shock is sufficient to decrease *P. berghei* infection without requiring a prerequisite bacterial immune challenge as reported by Dieme et al. (44). The resistance of *Anopheles* to *Plasmodium* infection induced by high temperature has been reported elsewhere (45), though the mechanism underlying the phenomenon was not described. Here we observed that thermal stress affected the transcription of both heat shock proteins and elements of the anti-pathogenic immune response. Given the recent increases in temperature in the tropical regions, the effect of heat shock on malaria transmission is relevant to future disease tends.

## Data Availability Statement

The datasets presented in this study can be found in online repositories. The names of the repository/repositories and accession number(s) can be found in the article/**Supplementary Material**.

## Author Contributions

RC and HL-M conceived the presented idea. RC developed the theory and performed the statistics. EH-T, FC-P, BR-T, KM-M, and VC-J carried out the experiments. RC and HL-M wrote the manuscript with support from BR-T and FC-P. FC-P, KM-M, and VC-J fabricated the mosquito samples. EH-T, FC-P, BR-T, KM-M, and VC-J processed and analyzed the samples. HL-M supervised the project and planned the experiments. All authors contributed to the article and approved the submitted version.

## Conflict of Interest

The authors declare that the research was conducted in the absence of any commercial or financial relationships that could be construed as a potential conflict of interest.
